# Supplementary material for: Oncolytic adenovirus expressing bispecific antibody targets T‐cell cytotoxicity in cancer biopsies
Source: EMBO Mol Med. 2017 Jun 20;9(8):1067–87. doi: 10.15252/emmm.201707567 (PMC5538299; doi:10.15252/emmm.201707567)
Supplement: Supplementary file 1 — Appendix [file EMMM-9-1067-s001.pdf]

Oncolytic adenovirus expressing bispecific antibody targets T cell cytotoxicity in cancer biopsies *(100 character limit including spaces)*

Joshua Freedman, Joachim Hagel, Ioannis Psallidas, Avinash Gupta, Laura Spiers, Paul Miller, Nikolaos Kanellakis, Rebecca Ashfield, Kerry D. Fisher, Margaret R. Duffy, Leonard W. Seymour

Appendix

Appendix Figure S1

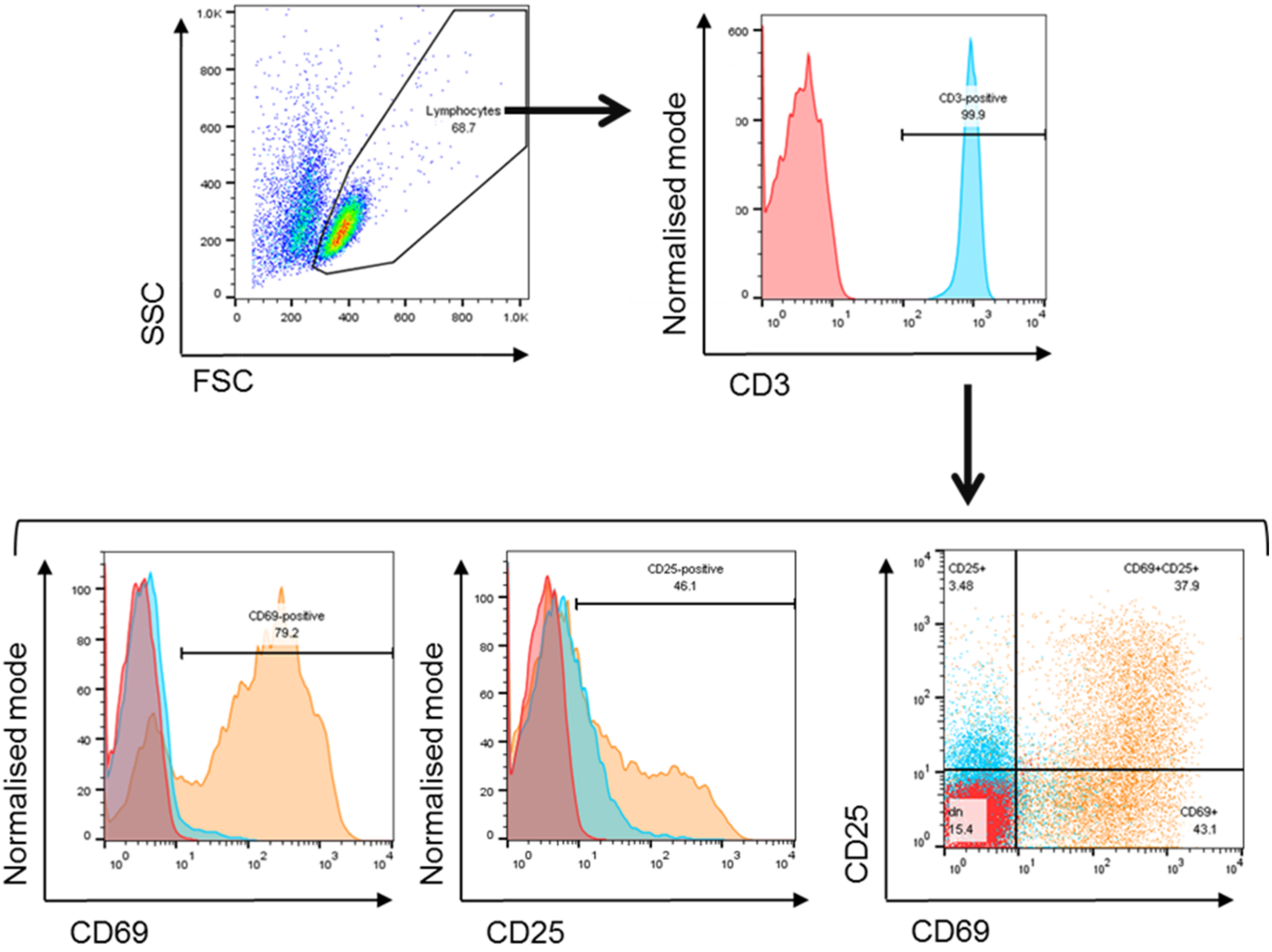

Appendix Figure S1: Example analysis of flow cytometry data

PBMC-purified T cells were stimulated with anti-CD3/CD28 beads or left untreated. After 24 h, cells were harvested and stained for CD3, CD69 and CD25. Cell debris was excluded by analysing FSC and SSC properties (top left panel). Within this gate, CD3-positive cells were selected (top right panel) for further analysis of their expression of activation markers CD69 and CD25 (bottom panels). Isotype controls for CD69 and CD25 were used to establish suitable gates (red). Increases in CD69 and CD25 expression were observed upon stimulation (orange) compared to untreated (blue).
