# Supplementary material for: Oncolytic adenovirus expressing bispecific antibody targets T‐cell cytotoxicity in cancer biopsies
Source: EMBO Mol Med. 2017 Jun 20;9(8):1067–87. doi: 10.15252/emmm.201707567 (PMC5538299; doi:10.15252/emmm.201707567)
Supplement: Supplementary file 2 — Expanded View Figures PDF [file EMMM-9-1067-s002.pdf]

Expanded View Figures

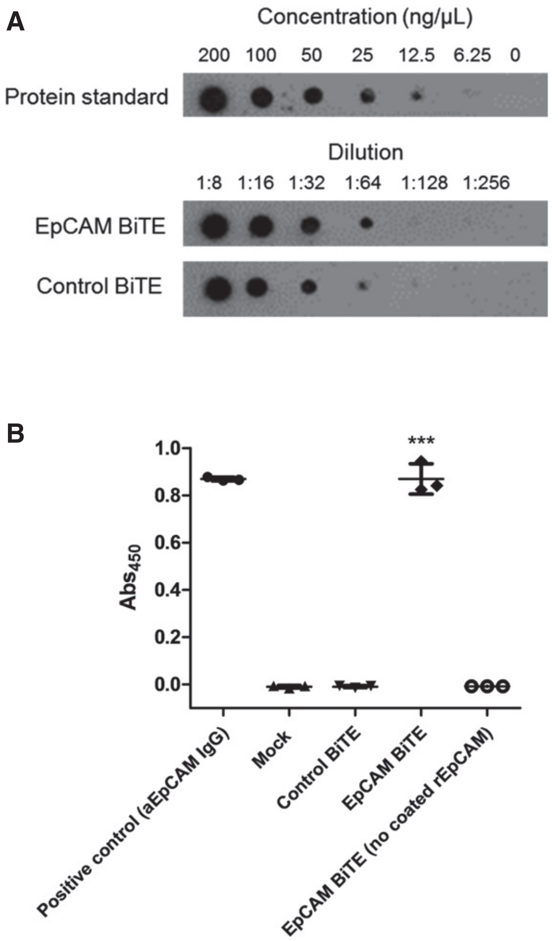

Figure EV1. Characterisation of recombinant EpCAM BiTE.

A Dot blot to estimate the quantity of EpCAM BiTE produced by transfected HEK293A cells.

B ELISA measuring the level of EpCAM binding by controls or recombinant EpCAM or non-specific BiTE. Each condition was measured in biological triplicate and represented as mean ± SD. Significance was assessed by comparison to empty vector control sample using a one-way ANOVA test with Tukey's *post hoc* analysis, ***$P < 0.001$.

Source data are available online for this figure.

© 2017 The Authors

A

	Virus titre (vp/mL)	Infectious particles (PFU/mL)	vp:PFU ratio
**EnAd**	4.64E+12	2.91E+ 11	15.9
**EnAd-CMV-ControlBiTE**	4.81E+11	1.00E+10	48.1
**EnAd-CMV-EpCAMBiTE**	1.00E+12	2.51E+10	39.9
**EnAd-SA-ControlBiTE**	1.61E+12	3.98E+10	40.6
**EnAd-SA-EpCAMBiTE**	1.22E+12	3.16E+10	38.7

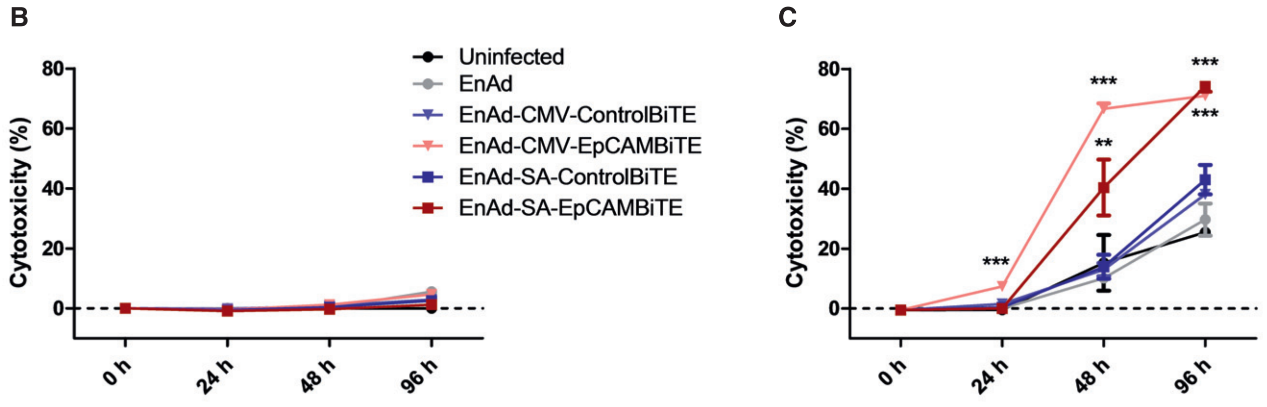

Figure EV2. Cytotoxicity of EnAd expressing EpCAM BiTE in SKOV3 cells.

A A quality control of viruses used in this study. The virus titre (vp/ml) was measured by PicoGreen assay and infectious particles (PFU/ml) by TCID50.

B, C SKOV3 cells were incubated with EnAd or recombinant viruses in the absence (B) or presence (C) of T cells and cytotoxicity was measured by LDH release at the specified time points. Each condition was measured in biological triplicate and represented as mean ± SD. Significance was assessed by comparison to uninfected control wells using a one-way ANOVA test with Tukey's *post hoc* analysis, $^{**}P < 0.01$, $^{***}P < 0.001$.

Source data are available online for this figure.

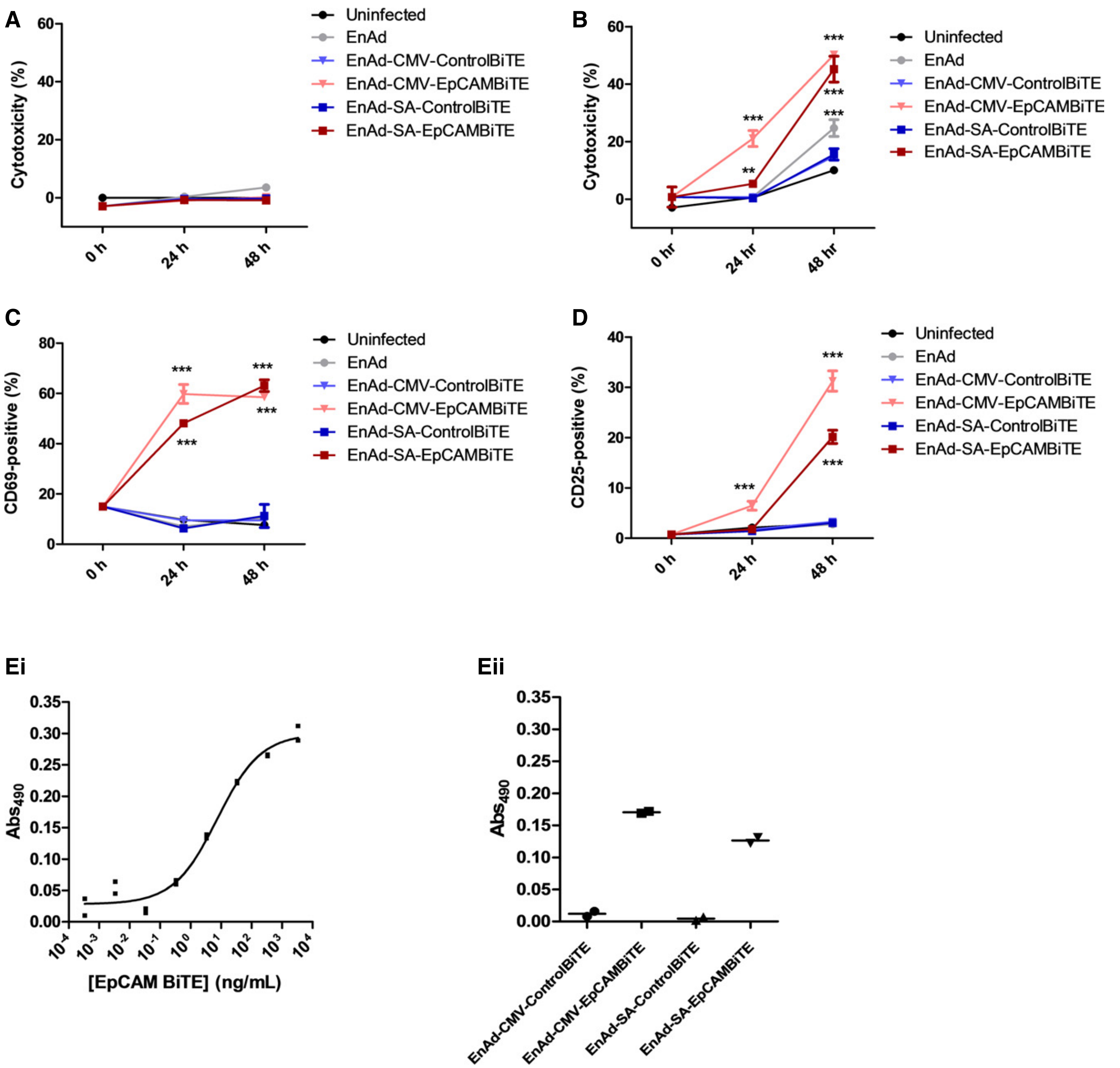

Figure EV3. Cytotoxicity and T-cell activation by EnAd expressing EpCAM BiTE in DLD cells.

A, B Cytotoxicity for infected DLD cells in the absence (A) or presence of T cells (B). DLD cells were infected and co-cultured with T cells, and cytotoxicity was measured by LDH release at the specified time points.

C, D T cells from (B) were harvested and stained for activation markers CD69 (C) or CD25 (D) and analysed via flow cytometry.

E Quantification of EpCAM BiTE produced from DLD cells infected with recombinant viruses. Standard curve of LDH released (Abs) of DLD cells in co-culture with $CD3^+$ cells and serial dilutions of a known quantity of recombinant EpCAM BiTE (Ei). In parallel, co-cultures were incubated with diluted supernatants (10,000-fold) from 3-day infected DLD cells (Eii). Standard curve allowed the approximate determination of EpCAM BiTE produced at 165 and 50 μg per million DLD cells for EnAd-CMV-EpCAMBiTE and EnAd-SA-EpCAMBiTE, respectively.

Data information: Each condition was measured in biological triplicate (A–D) or duplicate (E) and represented as mean ± SD (A–D). Significance was assessed by comparison to uninfected control wells using a one-way ANOVA test with Tukey's *post hoc* analysis, $^{**}P < 0.01$, $^{***}P < 0.001$.
Source data are available online for this figure.

 © 2017 The Authors

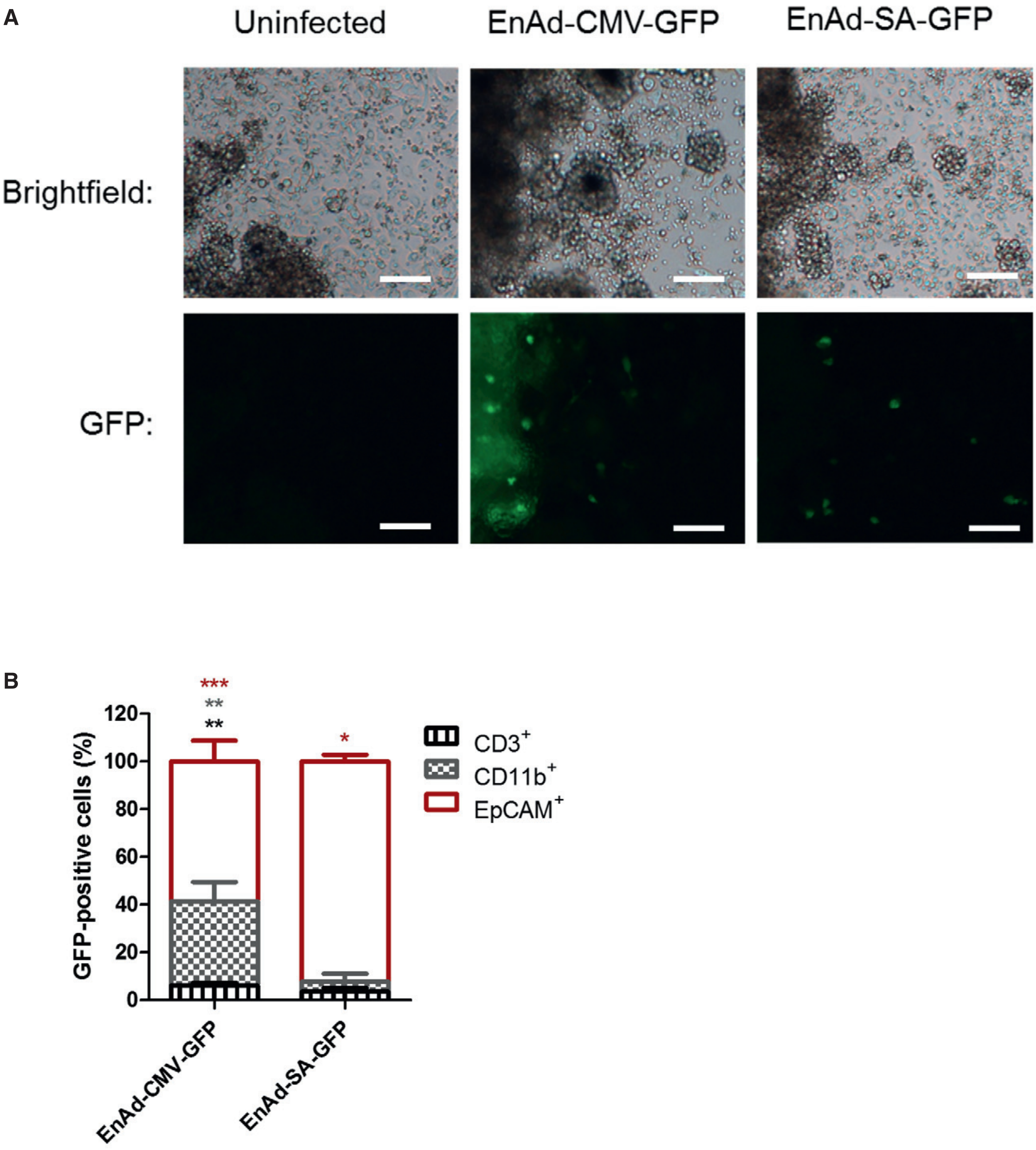

Figure EV4. Replication (SA promoter)-dependent GFP transgene is selectively expressed in $EpCAM^+$ tumour cells.

A, B Total cells from a peritoneal ascites sample were incubated with EnAd-CMV-GFP or EnAd-SA-GFP, with uninfected cells serving as a negative control. After 3 days, wells were imaged by bright-field or fluorescence microscopy. Original magnification ×10; scale bar, 100 μm (A). Total cells were harvested, and the proportion of GFP^+ cells that were CD3-, CD11b- or EpCAM-positive determined using flow cytometry (B). Each condition was measured in biological quadruplicate and represented as mean ± SD. Significance was assessed by comparison to uninfected control wells using a one-way ANOVA test with Tukey's *post hoc* analysis, $*P < 0.05$, $**P < 0.01$, $***P < 0.001$.

Source data are available online for this figure.

© 2017 The Authors

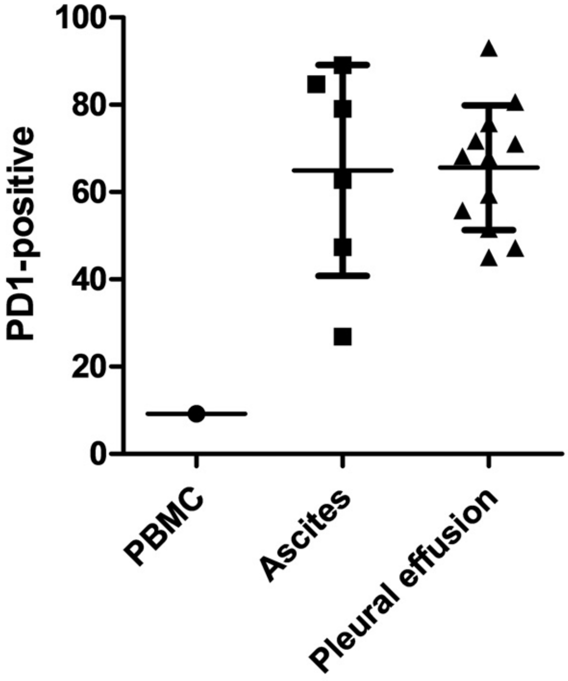

Figure EV5. Expression of PD-1 on PBMC and malignant exudate T cells.

The expression of PD-1 by endogenous T cells following their initial isolation from PBMC, ascites and pleural effusions was assessed by flow cytometry. PD-1 expression on CD3 cells within each donor sample was measured once and represented as mean ± SD of all measured samples.

Source data are available online for this figure.

 © 2017 The Authors
