# Supplementary material for: Oncolytic adenovirus expressing bispecific antibody targets T‐cell cytotoxicity in cancer biopsies
Source: EMBO Mol Med. 2017 Jun 20;9(8):1067–87. doi: 10.15252/emmm.201707567 (PMC5538299; doi:10.15252/emmm.201707567)
Supplement: Supplementary file 9 — Source Data for Expanded View [file EMMM-9-1067-s018.zip › Source_Data_for_Expanded_View_and_Appendix/Figure_EV1B.pdf]

	Abs$_{450}$		
	1	2	3
Positive control (aEpCAM IgG)	0.866093	0.87806	0.862779
Mock	-0.016539	-0.006344	-0.006767
Control BiTE	-0.014319	-0.006477	-0.007364
EpCAM BiTE	0.943238	0.840535	0.824582
EpCAM BiTE (no coated rEpCAM)	-0.00776	-0.008186	-0.007906
