# Supplementary material for: Oncolytic adenovirus expressing bispecific antibody targets T‐cell cytotoxicity in cancer biopsies
Source: EMBO Mol Med. 2017 Jun 20;9(8):1067–87. doi: 10.15252/emmm.201707567 (PMC5538299; doi:10.15252/emmm.201707567)
Supplement: Supplementary file 9 — Source Data for Expanded View [file EMMM-9-1067-s018.zip › Source_Data_for_Expanded_View_and_Appendix/Figure_EV2B.pdf]

Time (h)									Cytotoxicity (%)		
	Uninfected			EnAd			EnAd-CMV-ControlBiTE			EnAd-CMV-EpC/	
	1	2	3	1	2	3	1	2	3	1	2
0	0.00	0.00	0.00	0.00	0.00	0.00	0.00	0.00	0.00	0.00	0.00
24	0.00	0.00	0.00	0.63	-0.51	-0.13	-0.40	-0.23	0.26	-1.02	-0.68
48	0.00	0.00	0.00	-0.14	0.83	-0.06	0.78	1.09	0.62	0.41	2.09
96	0.00	0.00	0.00	6.86	6.10	3.68	2.88	3.73	2.24	4.00	5.86

AMBiTE	EnAd-SA-ControlBiTE			EnAd-SA-EpCAMBiTE		
3	1	2	3	1	2	3
0.00	0.00	0.00	0.00	0.00	0.00	0.00
0.36	-0.90	-0.71	-0.16	-1.29	-0.76	-0.59
1.12	0.06	0.19	1.00	0.17	-0.55	-0.55
4.16	3.02	3.86	0.92	2.49	1.10	0.14
