# Supplementary material for: Oncolytic adenovirus expressing bispecific antibody targets T‐cell cytotoxicity in cancer biopsies
Source: EMBO Mol Med. 2017 Jun 20;9(8):1067–87. doi: 10.15252/emmm.201707567 (PMC5538299; doi:10.15252/emmm.201707567)
Supplement: Supplementary file 9 — Source Data for Expanded View [file EMMM-9-1067-s018.zip › Source_Data_for_Expanded_View_and_Appendix/Figure_EV2C.pdf]

| Time (h) | | | | | | | | | Cytotoxicity (%) | | |
|---|---|---|---|---|---|---|---|---|---|---|---|
| | Uninfected | | | EnAd | | | EnAd-CMV-ControlBiTE | | | EnAd-CMV-EpC | |
| | 1 | 2 | 3 | 1 | 2 | 3 | 1 | 2 | 3 | 1 | 2 |
| 0 | -0.63 | -0.24 | -0.64 | -0.63 | -0.24 | -0.64 | -0.63 | -0.24 | -0.64 | -0.63 | -0.24 |
| 24 | 0.31 | -0.59 | -0.95 | 1.30 | -0.82 | -0.42 | 1.96 | 1.19 | 1.38 | 8.89 | 6.98 |
| 48 | 18.86 | 22.21 | 4.64 | 11.40 | 10.07 | 8.95 | 15.48 | 11.33 | 12.19 | 64.75 | 68.18 |
| 96 | 24.74 | 26.46 | 25.05 | 31.61 | 33.83 | 23.63 | 39.07 | 39.03 | 36.35 | 72.29 | 69.42 |

| AMBiTE | EnAd-SA-ControlBiTE | | | EnAd-SA-EpCAMBiTE | | |
|---|---|---|---|---|---|---|
| 3 | 1 | 2 | 3 | 1 | 2 | 3 |
| -0.64 | -0.63 | -0.24 | -0.64 | -0.63 | -0.24 | -0.64 |
| 6.12 | -0.25 | 0.54 | 0.90 | -0.11 | -0.67 | 0.74 |
| 67.15 | 11.03 | 18.47 | 12.19 | 49.54 | 30.88 | 40.69 |
| 71.48 | 46.27 | 45.40 | 37.38 | 73.65 | 76.14 | 72.74 |
