# Supplementary material for: Oncolytic adenovirus expressing bispecific antibody targets T‐cell cytotoxicity in cancer biopsies
Source: EMBO Mol Med. 2017 Jun 20;9(8):1067–87. doi: 10.15252/emmm.201707567 (PMC5538299; doi:10.15252/emmm.201707567)
Supplement: Supplementary file 9 — Source Data for Expanded View [file EMMM-9-1067-s018.zip › Source_Data_for_Expanded_View_and_Appendix/Figure_EV3A.pdf]

Time (h)						Cytotoxicity (%)				
	Uninfected			EnAd			EnAd-CMV-ControlBiTE			EnAd-CMV-EpC
	1	2	3	1	2	3	1	2	3	1	2
0	0.00	0.00	0.00	-3.28	-2.39	-3.14	-3.28	-2.39	-3.14	-3.28	-2.39
24	0.00	0.00	0.00	0.77	0.45	-0.10	-0.24	-0.25	-0.45	-1.10	-1.19
48	0.00	0.00	0.00	4.53	3.91	2.33	-0.74	-0.31	-0.88	0.00	0.21

AMBiTE	EnAd-SA-ControlBiTE			EnAd-SA-EpCAMBiTE		
3	1	2	3	1	2	3
-3.14	-3.28	-2.39	-3.14	-3.28	-2.39	-3.14
-0.65	-0.72	-0.23	-0.54	-0.84	-0.75	-0.61
0.47	-0.79	-0.11	0.18	-1.44	-0.56	-0.57
