# Supplementary material for: Oncolytic adenovirus expressing bispecific antibody targets T‐cell cytotoxicity in cancer biopsies
Source: EMBO Mol Med. 2017 Jun 20;9(8):1067–87. doi: 10.15252/emmm.201707567 (PMC5538299; doi:10.15252/emmm.201707567)
Supplement: Supplementary file 9 — Source Data for Expanded View [file EMMM-9-1067-s018.zip › Source_Data_for_Expanded_View_and_Appendix/Figure_EV3B.pdf]

Time (h)							Cytotoxicity (%)				
	Uninfected			EnAd			EnAd-CMV-ControlBiTE			EnAd-CMV-EpC/	
	1	2	3	1	2	3	1	2	3	1	2
0	-3.276	-2.386	-3.143	-3.276	2.3856	3.1427	-3.276	2.3856	3.1427	-3.276	2.3856
24	1.2432	-0.246	0.7648	0.6296	0.8337	0.8182	0.347	1.1243	0.622	17.903	22.566
48	11.608	9.2946	9.358	27.545	24.96	21.739	15.506	15.501	13.758	50.18	50.944

AMBiTE	EnAd-SA-ControlBiTE			EnAd-SA-EpCAMBiTE		
3	1	2	3	1	2	3
3.1427	-3.276	2.3856	3.1427	-3.276	2.3856	3.1427
22.824	0.6371	0.1323	0.281	5.9938	5.3036	4.7498
49.522	14.783	14.122	17.82	44.738	49.929	40.97
