# Supplementary material for: Oncolytic adenovirus expressing bispecific antibody targets T‐cell cytotoxicity in cancer biopsies
Source: EMBO Mol Med. 2017 Jun 20;9(8):1067–87. doi: 10.15252/emmm.201707567 (PMC5538299; doi:10.15252/emmm.201707567)
Supplement: Supplementary file 9 — Source Data for Expanded View [file EMMM-9-1067-s018.zip › Source_Data_for_Expanded_View_and_Appendix/Figure_EV3C.pdf]

Time (h)									CD69-positive (%)		
	Uninfected			EnAd			EnAd-CMV-ControlBiTE			EnAd-CMV-EpC[illegible]	
	1	2	3	1	2	3	1	2	3	1	2
0	14.8	14.9	15.1	14.8	14.9	15.1	14.8	14.9	15.1	14.8	14.9
24	8.88	10.5	9.68	6.24	8.45	6.5	9.8	10.3	8.45	55.5	62.7
48	8.24	7.28	7.4	10.8	10.2	10.1	8.81	10.1	9.55	58.2	57.9

AMBiTE	EnAd-SA-ControlBiTE			EnAd-SA-EpCAMBiTE		
3	1	2	3	1	2	3
15.1	14.8	14.9	15.1	14.8	14.9	15.1
61.2	6.63	5.36	6.95	47.9	47	49.4
59.4	9.19	8.04	16.5	61.9	65.8	61.6
