# Supplementary material for: Oncolytic adenovirus expressing bispecific antibody targets T‐cell cytotoxicity in cancer biopsies
Source: EMBO Mol Med. 2017 Jun 20;9(8):1067–87. doi: 10.15252/emmm.201707567 (PMC5538299; doi:10.15252/emmm.201707567)
Supplement: Supplementary file 9 — Source Data for Expanded View [file EMMM-9-1067-s018.zip › Source_Data_for_Expanded_View_and_Appendix/Figure_EV3D.pdf]

Time (h)									CD25-positive (%)		
	Uninfected			EnAd			EnAd-CMV-ControlBiTE			EnAd-CMV-EpC/	
	1	2	3	1	2	3	1	2	3	1	2
0	0.75	0.83	0.65	0.75	0.83	0.65	0.75	0.83	0.65	0.75	0.83
24	2.12	2.11	2.09	1.7	2.03	1.33	2.07	1.75	1.43	7.5	5.94
48	2.61	2.87	3.13	3.13	2.89	2.35	3	3.39	3.46	33.5	30.7

AMBiTE	EnAd-SA-ControlBiTE			EnAd-SA-EpCAMBiTE		
3	1	2	3	1	2	3
0.65	0.75	0.83	0.65	0.75	0.83	0.65
5.97	1.55	1.31	1.41	1.52	1.92	1.93
29.6	3.03	2.71	3.44	19.9	19	21.6
