# Supplementary material for: Oncolytic adenovirus expressing bispecific antibody targets T‐cell cytotoxicity in cancer biopsies
Source: EMBO Mol Med. 2017 Jun 20;9(8):1067–87. doi: 10.15252/emmm.201707567 (PMC5538299; doi:10.15252/emmm.201707567)
Supplement: Supplementary file 9 — Source Data for Expanded View [file EMMM-9-1067-s018.zip › Source_Data_for_Expanded_View_and_Appendix/Figure_EV3Ei.pdf]

EpCAM BiTE (ng/mL)	**Abs_{490}**			
	Control BiTE		EpCAM BiTE	
	1	2	1	2
3333.333	0.054	0.045	0.289	0.312
333.3333	0.013	0.013	0.266	0.264
33.33333	0.007	-0.009	0.221	0.224
3.333333	-0.01	-0.005	0.139	0.133
0.3333333	-0.004	-0.007	0.06	0.066
0.03333333	-0.003	-0.004	0.014	0.021
0.003333333	0.032	0.028	0.045	0.064
0.000333333	0.017	0.021	0.037	0.01
0	-0.02	-0.01	0.004	0.002
