# Supplementary material for: Oncolytic adenovirus expressing bispecific antibody targets T‐cell cytotoxicity in cancer biopsies
Source: EMBO Mol Med. 2017 Jun 20;9(8):1067–87. doi: 10.15252/emmm.201707567 (PMC5538299; doi:10.15252/emmm.201707567)
Supplement: Supplementary file 9 — Source Data for Expanded View [file EMMM-9-1067-s018.zip › Source_Data_for_Expanded_View_and_Appendix/Figure_EV3Eii.pdf]

**Treatment**	**Abs_{490}**	
	1	2
EnAd-CMV-ControlBiTE	0.016	0.008
EnAd-CMV-EpCAMBiTE	0.172	0.169
EnAd-SA-ControlBiTE	0.007	0.002
EnAd-SA-EpCAMBiTE	0.131	0.122
