# Supplementary material for: Oncolytic adenovirus expressing bispecific antibody targets T‐cell cytotoxicity in cancer biopsies
Source: EMBO Mol Med. 2017 Jun 20;9(8):1067–87. doi: 10.15252/emmm.201707567 (PMC5538299; doi:10.15252/emmm.201707567)
Supplement: Supplementary file 9 — Source Data for Expanded View [file EMMM-9-1067-s018.zip › Source_Data_for_Expanded_View_and_Appendix/Figure_EV4B.pdf]

		GFP-positive cells (%)		
		CD3+	CD11b+	EpCAM+
EnAd-CMV-GFP	1	5.54	31.57	62.89
	2	6.46	27.67	65.87
	3	7.25	48.75	44.00
	4	4.31	32.68	63.01
	5	7.00	36.12	56.88
EnAd-SA-GFP	1	1.86	5.74	92.40
	2	2.09	8.26	89.65
	3	3.30	1.60	95.10
	4	5.40	5.40	89.20
	5	4.88	0.00	95.12
