# Supplementary material for: Oncolytic adenovirus expressing bispecific antibody targets T‐cell cytotoxicity in cancer biopsies
Source: EMBO Mol Med. 2017 Jun 20;9(8):1067–87. doi: 10.15252/emmm.201707567 (PMC5538299; doi:10.15252/emmm.201707567)
Supplement: Supplementary file 9 — Source Data for Expanded View [file EMMM-9-1067-s018.zip › Source_Data_for_Expanded_View_and_Appendix/Figure_EV5.pdf]

PD1-positive cells (%)

PBMC	Ascites	Pleural effusion
9.18	47.4	45
	62.8	59.4
	26.9	47.2
	79	75.7
	89	93
	84.7	71
		71.7
		80.6
		68.2
		67.9
		51.5
		55.8
