# Supplementary figures and images for: Oncolytic adenovirus expressing bispecific antibody targets T‐cell cytotoxicity in cancer biopsies
Source: EMBO Mol Med. 2017 Jun 20;9(8):1067–87. doi: 10.15252/emmm.201707567 (PMC5538299; doi:10.15252/emmm.201707567)

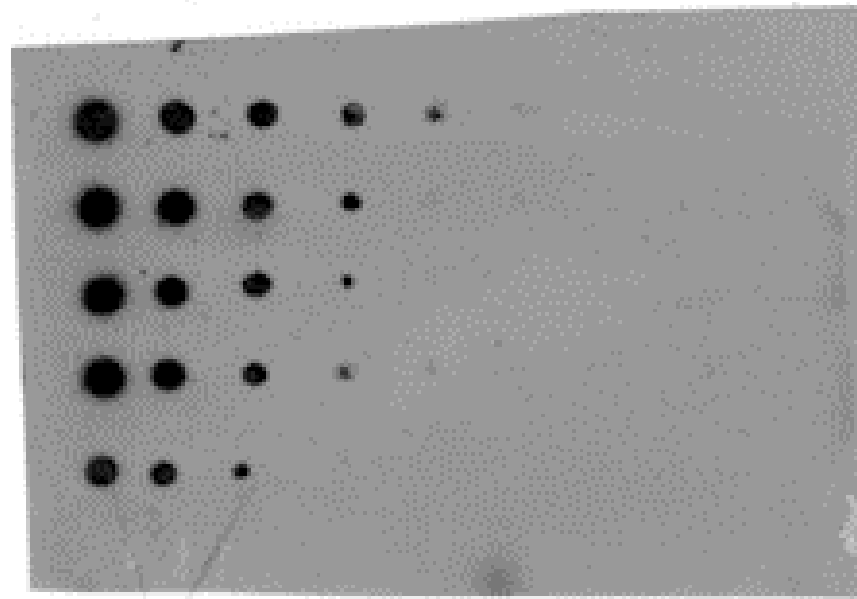

Supplement: Supplementary file 9 — Source Data for Expanded View [file EMMM-9-1067-s018.zip › Source_Data_for_Expanded_View_and_Appendix/Figure_EV1A.pdf]

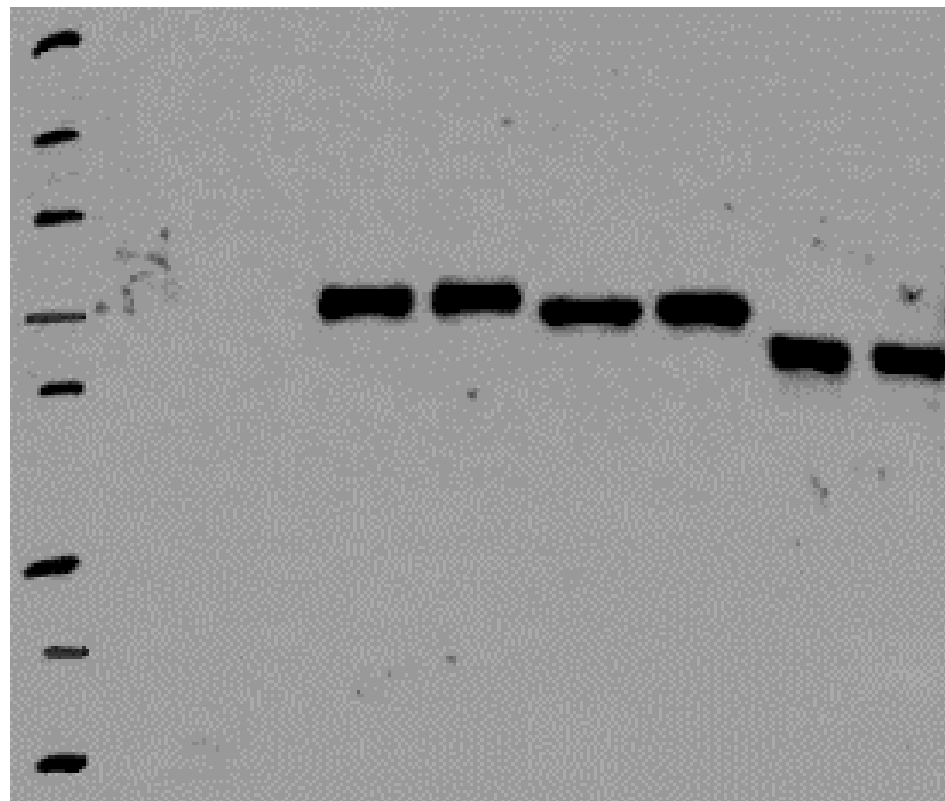

Supplement: Supplementary file 14 — Source Data for Figure 4 [file EMMM-9-1067-s012.zip › EMM_07567_Fig4_Source_data/Fig4D.pdf]

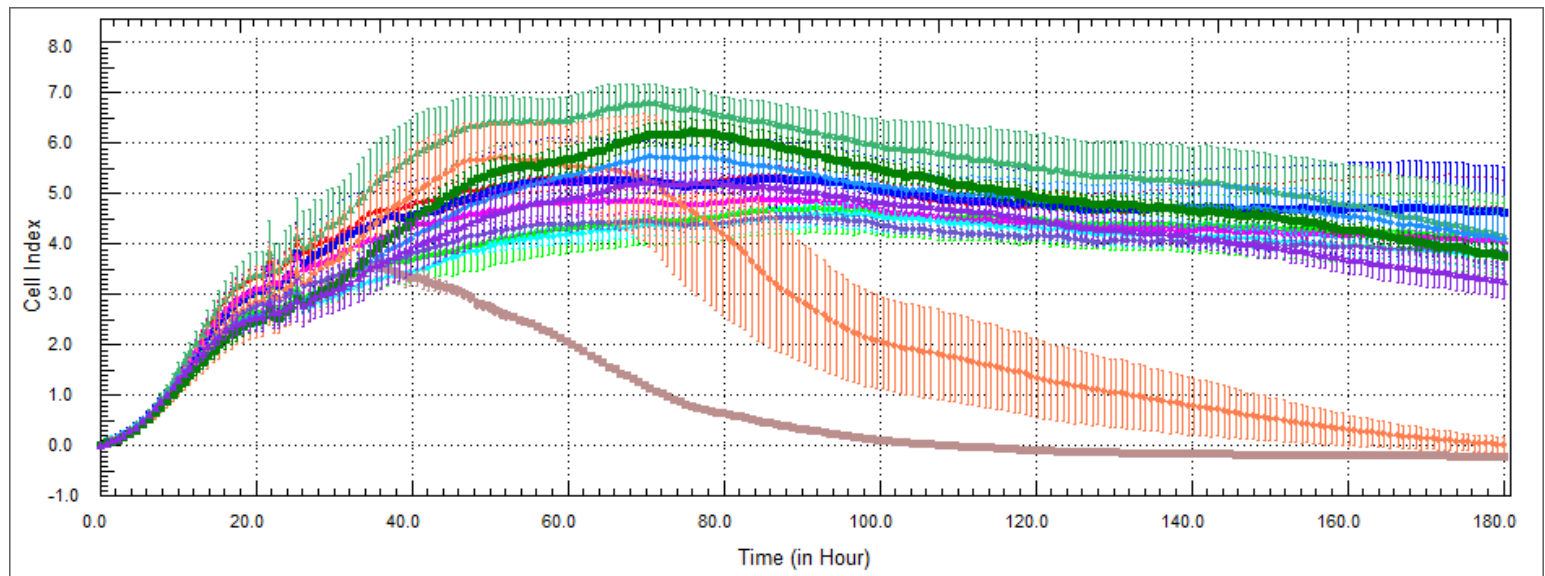

Supplement: Supplementary file 15 — Source Data for Figure 5 [file EMMM-9-1067-s013.zip › EMM_07567_Fig5_Source_data/Fig5AB.pdf]

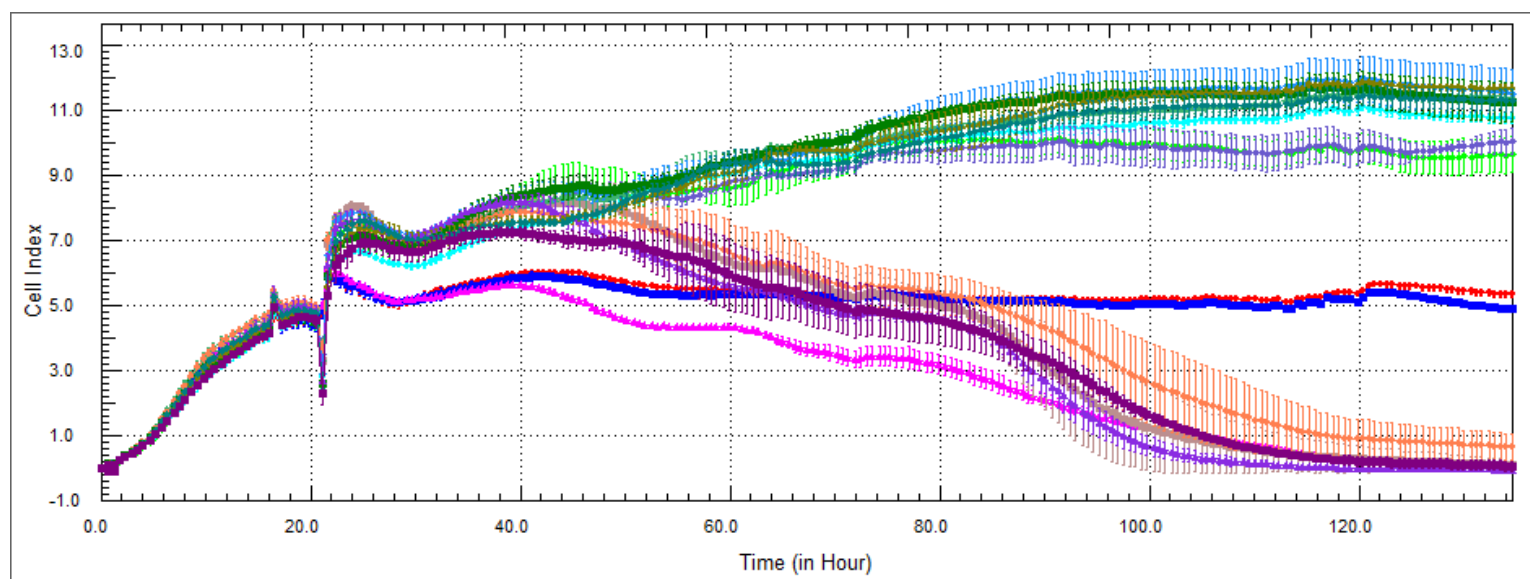

Supplement: Supplementary file 17 — Source Data for Figure 7 [file EMMM-9-1067-s015.zip › EMM_07567_Fig7_Source_data/Fig7F.pdf]
