# Supplementary material for: Oncolytic adenovirus expressing bispecific antibody targets T‐cell cytotoxicity in cancer biopsies
Source: EMBO Mol Med. 2017 Jun 20;9(8):1067–87. doi: 10.15252/emmm.201707567 (PMC5538299; doi:10.15252/emmm.201707567)
Supplement: Supplementary file 11 — Source Data for Figure 1 [file EMMM-9-1067-s009.zip › EMM_07567_Fig1_Source_data/Fig1B.pdf]

	CD69-positive (%)			CD25-positive (%)		
**Treatment**	1	2	3	1	2	3
IgG isotype	8.76	5.7	6.04	27.3	26.5	26.7
aCD3/28	80.9	80.9	80.5	45.5	45.2	44.8
Untreated	5.61	5.76	5.41	27.6	27.6	27.8
Control BiTE	6.59	6.11	7.14	26.6	26.7	25.7
EpCAM BiTE	13.1	14.8	13.9	29.6	27.2	29.8
DLD	5.55	5.29	5.39	27.2	27.2	27
Control BiTE + DLD	5.74	6.07	5.88	25.7	26.6	25.7
EpCAM BiTE + DLD	85.4	86.7	86.4	67.6	68.6	68.5
