# Supplementary material for: Oncolytic adenovirus expressing bispecific antibody targets T‐cell cytotoxicity in cancer biopsies
Source: EMBO Mol Med. 2017 Jun 20;9(8):1067–87. doi: 10.15252/emmm.201707567 (PMC5538299; doi:10.15252/emmm.201707567)
Supplement: Supplementary file 11 — Source Data for Figure 1 [file EMMM-9-1067-s009.zip › EMM_07567_Fig1_Source_data/Fig1C.pdf]

	IFNγ-positive (%)		
**Treatment**	1	2	3
Untreated	1.08	0.86	0.78
PMA/Ionomycin	30.2	27.6	27.9
DLD	0.4	0.57	0.39
Control BiTE + DLD	0.39	0.28	0.51
EpCAM BiTE + DLD	6.84	6.71	7.02
