# Supplementary material for: Oncolytic adenovirus expressing bispecific antibody targets T‐cell cytotoxicity in cancer biopsies
Source: EMBO Mol Med. 2017 Jun 20;9(8):1067–87. doi: 10.15252/emmm.201707567 (PMC5538299; doi:10.15252/emmm.201707567)
Supplement: Supplementary file 11 — Source Data for Figure 1 [file EMMM-9-1067-s009.zip › EMM_07567_Fig1_Source_data/Fig1D.pdf]

	Division index			Proliferating T-cells (%)		
**Treatment**	1	2	3	1	2	3
Untreated	0.116	0.000114	0	1.61	2.48	3.79
aCD3/28	1.62	1.43	1.01	58.7	69	83
DLD	0.0847	0.0728	0.0685	13.6	10.8	11.4
Control BiTE + DLD	0.0956	0.1	0.0796	12.5	10	12.8
EpCAM BiTE + DLD	1.29	1.15	1.31	64	50	57.6
