# Supplementary material for: Oncolytic adenovirus expressing bispecific antibody targets T‐cell cytotoxicity in cancer biopsies
Source: EMBO Mol Med. 2017 Jun 20;9(8):1067–87. doi: 10.15252/emmm.201707567 (PMC5538299; doi:10.15252/emmm.201707567)
Supplement: Supplementary file 11 — Source Data for Figure 1 [file EMMM-9-1067-s009.zip › EMM_07567_Fig1_Source_data/Fig1E.pdf]

	CD107a-positive (%)		
**Treatment**	1	2	3
Untreated	1.8	1.22	1.52
aCD3/28	17.6	16.6	17.1
DLD	1	0.97	1.12
Control BiTE + DLD	2.1	1.95	1.66
EpCAM BiTE + DLD	19.6	17.4	16.7
