# Supplementary material for: Oncolytic adenovirus expressing bispecific antibody targets T‐cell cytotoxicity in cancer biopsies
Source: EMBO Mol Med. 2017 Jun 20;9(8):1067–87. doi: 10.15252/emmm.201707567 (PMC5538299; doi:10.15252/emmm.201707567)
Supplement: Supplementary file 11 — Source Data for Figure 1 [file EMMM-9-1067-s009.zip › EMM_07567_Fig1_Source_data/Fig1F.pdf]

Cytokine (pg/mL)	Treatment								
	DLD			DLD + Control BiTE			DLD + EpCAM BiTE		
IL-5	2.92	3.1	<2.91	2.98	3.04	<2.91	6.73	6.34	13.49
IL-13	<3.41	<3.41	<3.41	<3.41	<3.41	<3.41	11.46	24.39	42.03
IL-2	20.44	12.16	9.15	17.57	27.93	11.64	2186.88	2244.51	4033.41
IL-6	78.67	83.76	50.83	96.03	83.76	25.79	877.15	675.8	1415.75
IL-9	<3.70	3.89	<3.70	4.47	<3.70	<3.70	7.4	17.81	24.9
IL-10	6.21	9.85	6.31	7.68	10.2	6.53	50.21	468.86	972.01
IFNg	493.19	186.05	302.33	805.48	1421.34	797.02	11321.98	26447.93	26447.93
TNFa	6.8	7.17	7.17	18.29	10.84	8.71	377.87	1052.86	848.74
IL-17A	4.37	5.21	<4.19	5.14	5.29	<4.19	9.91	14.08	20.26
IL-17F	<3.49	<3.49	<3.49	<3.49	<3.49	<3.49	11.25	42.28	61.12
IL-4	<4.76	<4.76	<4.76	<4.76	<4.76	<4.76	5.25	7.96	6.49
IL-21	7.66	8.23	6.93	8.63	7.47	<6.70	33.98	73.54	62.49
IL-22	8.9	9.45	8.1	9.17	9.45	8.1	15.2	29.08	32.79

Cytokine (fold)	Treatment								
	DLD			DLD + Control BiTE			DLD + EpCAM BiTE		
IL-5	0.98	1.04	0.98	1.00	1.02	0.98	2.26	2.13	4.53
IL-13	1.00	1.00	1.00	1.00	1.00	1.00	3.36	7.15	12.33
IL-2	1.47	0.87	0.66	1.26	2.01	0.84	157.10	161.24	289.76
IL-6	1.11	1.18	0.72	1.35	1.18	0.36	12.34	9.51	19.91
IL-9	0.98	1.03	0.98	1.19	0.98	0.98	1.97	4.74	6.62
IL-10	0.83	1.32	0.85	1.03	1.37	0.88	6.73	62.85	130.30
IFNg	1.51	0.57	0.92	2.46	4.34	2.44	34.60	80.83	80.83
TNFa	0.96	1.02	1.02	2.59	1.54	1.24	53.60	149.34	120.39
IL-17A	0.91	0.91	0.91	1.07	1.10	0.87	2.07	2.94	4.23
IL-17F	1.00	1.00	1.00	1.00	1.00	1.00	3.22	12.11	17.51
IL-4	1.00	1.00	1.00	1.00	1.00	1.00	1.10	1.67	1.36
IL-21	1.01	1.08	0.91	1.13	0.98	0.88	4.47	9.66	8.21
IL-22	1.01	1.07	0.92	1.04	1.07	0.92	1.72	3.30	3.72
