# Supplementary material for: Oncolytic adenovirus expressing bispecific antibody targets T‐cell cytotoxicity in cancer biopsies
Source: EMBO Mol Med. 2017 Jun 20;9(8):1067–87. doi: 10.15252/emmm.201707567 (PMC5538299; doi:10.15252/emmm.201707567)
Supplement: Supplementary file 12 — Source Data for Figure 2 [file EMMM-9-1067-s010.zip › EMM_07567_Fig2_Source_data/Fig2A.pdf]

Treatment	Cytotoxicity (%)					
	CHO			CHO-EpCAM		
	1	2	3	1	2	3
Control BiTE	-0.04392	1.795182	0.483937	0.449	-0.235	0.295
EpCAM BiTE	1.053252	1.385525	1.268166	-0.379	0.494	0.494
T-cells	0.419287	0.55905	-0.489168	3.813	2.542	1.986
Control BiTE + T-cells	0.209644	1.257862	0.908456	2.859	0.635	3.018
EpCAM BiTE + T-cells	1.677149	0.838574	1.257862	46.783	43.685	44.718
