# Supplementary material for: Oncolytic adenovirus expressing bispecific antibody targets T‐cell cytotoxicity in cancer biopsies
Source: EMBO Mol Med. 2017 Jun 20;9(8):1067–87. doi: 10.15252/emmm.201707567 (PMC5538299; doi:10.15252/emmm.201707567)
Supplement: Supplementary file 12 — Source Data for Figure 2 [file EMMM-9-1067-s010.zip › EMM_07567_Fig2_Source_data/Fig2B.pdf]

| Treatment | CD25-positive (%) | | | | | |
|---|---|---|---|---|---|---|
| | CHO | | | CHO-EpCAM | | |
| | 1 | 2 | 3 | 1 | 2 | 3 |
| Untreated | 3.15 | 2.83 | 3.12 | 2.65 | 3.11 | 3.07 |
| Control BiTE | 3.05 | 2.78 | 2.77 | 3.11 | 2.71 | 3.14 |
| EpCAM BiTE | 4.84 | 5.32 | 5.38 | 50.5 | 53.2 | 50.3 |
