# Supplementary material for: Oncolytic adenovirus expressing bispecific antibody targets T‐cell cytotoxicity in cancer biopsies
Source: EMBO Mol Med. 2017 Jun 20;9(8):1067–87. doi: 10.15252/emmm.201707567 (PMC5538299; doi:10.15252/emmm.201707567)
Supplement: Supplementary file 12 — Source Data for Figure 2 [file EMMM-9-1067-s010.zip › EMM_07567_Fig2_Source_data/Fig2C.pdf]

**Cell line**	**Cytotoxicity (%)**								
	Untreated			Control BiTE			EpCAM BiTE		
	1	2	3	1	2	3	1	2	3
DLD	13.771	6.224	16.73	10.21	7.721	13.732	86.183	77.725	79.286
HT-29	14.279	11.277	19.796	24.27	18.804	11.559	52.416	53.932	54.996
SKOV3	-2.456	-4.533	1.731	2.255	-0.323	13.042	47.765	51.018	50.316
A431	21.263	18.781	20.06	12.154	15.593	13.004	57.99	63.145	61.971
A549	-16.594	-12.764		-10.847	-9.161		40.183	38.353	50.316
PC3	3.532	7.845	-2.487	-3.555	0.751	2.372	27.636	39.262	41.138
