# Supplementary material for: Oncolytic adenovirus expressing bispecific antibody targets T‐cell cytotoxicity in cancer biopsies
Source: EMBO Mol Med. 2017 Jun 20;9(8):1067–87. doi: 10.15252/emmm.201707567 (PMC5538299; doi:10.15252/emmm.201707567)
Supplement: Supplementary file 12 — Source Data for Figure 2 [file EMMM-9-1067-s010.zip › EMM_07567_Fig2_Source_data/Fig2D.pdf]

Cell line	EpCAM (gMFI)	
	Isotype	EpCAM
DLD	1.81	361
HT-29	1.6	311
SKOV3	2	103
A431	2.07	82.1
A549	2.11	18.2
PC3	1.82	5.8
