# Supplementary material for: Oncolytic adenovirus expressing bispecific antibody targets T‐cell cytotoxicity in cancer biopsies
Source: EMBO Mol Med. 2017 Jun 20;9(8):1067–87. doi: 10.15252/emmm.201707567 (PMC5538299; doi:10.15252/emmm.201707567)
Supplement: Supplementary file 13 — Source Data for Figure 3 [file EMMM-9-1067-s011.zip › EMM_07567_Fig3_Source_data/Fig3A.pdf]

Subset	CD69-positive (%)						CD25-positive (%)					
	Control BiTE			EpCAM BiTE			Control BiTE			EpCAM BiTE		
	1	2	3	1	2	3	1	2	3	1	2	3
CD4	1.21	1.23	1.22	71.5	68.6	64.4	21.1	22.7	20.8	52.9	56.5	52.2
CD8	8.59	7.97	7.74	60.5	61.2	56.2	9.7	9.59	9.03	33.4	33.6	36.1
