# Supplementary material for: Oncolytic adenovirus expressing bispecific antibody targets T‐cell cytotoxicity in cancer biopsies
Source: EMBO Mol Med. 2017 Jun 20;9(8):1067–87. doi: 10.15252/emmm.201707567 (PMC5538299; doi:10.15252/emmm.201707567)
Supplement: Supplementary file 13 — Source Data for Figure 3 [file EMMM-9-1067-s011.zip › EMM_07567_Fig3_Source_data/Fig3C.pdf]

**Subset**	**CD107a-positive (%)**					
	Control BiTE			EpCAM BiTE		
	1	2	3	1	2	3
CD4	2.31	2.08	2.73	30.5	33.1	32.1
CD8	1.15	1.31	1.17	27.1	27.9	28.1
