# Supplementary material for: Oncolytic adenovirus expressing bispecific antibody targets T‐cell cytotoxicity in cancer biopsies
Source: EMBO Mol Med. 2017 Jun 20;9(8):1067–87. doi: 10.15252/emmm.201707567 (PMC5538299; doi:10.15252/emmm.201707567)
Supplement: Supplementary file 13 — Source Data for Figure 3 [file EMMM-9-1067-s011.zip › EMM_07567_Fig3_Source_data/Fig3D.pdf]

Subset	Cytotoxicity (%)					
	Control BiTE			EpCAM BiTE		
	1	2	3	1	2	3
CD4	-0.163	-0.433	0.129	15.62	14.8	16.57
CD8	-1.601	-1.553	-1.146	8.265	9.936	11.5
