# Supplementary material for: Oncolytic adenovirus expressing bispecific antibody targets T‐cell cytotoxicity in cancer biopsies
Source: EMBO Mol Med. 2017 Jun 20;9(8):1067–87. doi: 10.15252/emmm.201707567 (PMC5538299; doi:10.15252/emmm.201707567)
Supplement: Supplementary file 14 — Source Data for Figure 4 [file EMMM-9-1067-s012.zip › EMM_07567_Fig4_Source_data/Fig4B.pdf]

Treatment	Genomes (/mL)					
	24 hr			72 hr		
	1	2	3	1	2	3
Uninfected	128060.6	557283.7	719505.4	42318210	37134900	7340365
EnAd	6.179E+11	7.036E+11	7.522E+11	5.49E+12	5.372E+12	6.151E+12
EnAd-CMV-ControlBiTE	7.028E+11	7.042E+11	5.572E+11	4.552E+12	4.986E+12	4.074E+12
EnAd-SA-ControlBiTE	7.309E+11	9.888E+11	8.59E+11	5E+12	4.905E+12	5.506E+12
EnAd-CMV-EpCAMBiTE	7.379E+11	5.801E+11	6.395E+11	4.282E+12	4.125E+12	3.193E+12
EnAd-SA-EpCAMBiTE	1.13E+12	1.215E+12	1.541E+12	4.984E+12	5.08E+12	4.312E+12
