# Supplementary material for: Oncolytic adenovirus expressing bispecific antibody targets T‐cell cytotoxicity in cancer biopsies
Source: EMBO Mol Med. 2017 Jun 20;9(8):1067–87. doi: 10.15252/emmm.201707567 (PMC5538299; doi:10.15252/emmm.201707567)
Supplement: Supplementary file 14 — Source Data for Figure 4 [file EMMM-9-1067-s012.zip › EMM_07567_Fig4_Source_data/Fig4C.pdf]

vp/cell							Treatment	
		EnAd			EnAd-CMV-ControlBiTE		EnAd-CMV-EpCA[illegible]	
	1	2	3	1	2	3	1	2
0.000256	83.67	82.38	91.28	85.76	95.26	83.54	86.37	90.32
0.00128	106.97	89.54	94.08	82.55	82.22	76.15	72.23	74.73
0.0064	114.75	79.93	81.10	67.17	80.42	67.47	76.78	68.80
0.032	85.44	63.83	72.92	60.94	58.95	54.34	50.25	48.94
0.16	36.14	47.77	52.09	33.41	42.93	26.74	33.61	33.63
0.8	29.08	23.44	25.21	24.17	21.75	13.55	18.49	15.77
4	5.26	0.45	1.05	7.88	9.13	1.51	4.64	2.22
20	3.64	-1.99	-5.51	5.69	6.40	4.41	14.40	4.16

MBiTE	EnAd-SA-ControlBiTE			EnAd-SA-EpCAMBiTE		
3	1	2	3	1	2	3
80.91	88.47	102.58	88.78	114.39	118.36	81.25
96.04	77.26	87.44	73.12	100.67	78.02	76.92
81.64	71.54	80.03	79.07	84.14	87.28	70.75
37.55	50.27	73.12	68.47	55.79	58.84	54.31
30.50	34.71	38.39	45.71	17.65	29.12	38.16
12.56	7.63	23.77	24.88	1.75	10.83	22.25
3.05	1.27	8.54	9.30	-3.21	6.61	8.91
1.13	-0.70	4.00	6.22	-1.38	7.44	12.98
