# Supplementary material for: Oncolytic adenovirus expressing bispecific antibody targets T‐cell cytotoxicity in cancer biopsies
Source: EMBO Mol Med. 2017 Jun 20;9(8):1067–87. doi: 10.15252/emmm.201707567 (PMC5538299; doi:10.15252/emmm.201707567)
Supplement: Supplementary file 14 — Source Data for Figure 4 [file EMMM-9-1067-s012.zip › EMM_07567_Fig4_Source_data/Fig4E.pdf]

Treatment	CD25-positive (%)					
	CHO			CHO-EpCAM		
	1	2	3	1	2	3
Uninfected	8.85	8.73	9.26	7.84	8.23	7.78
EnAd	8.07	9.26	9.09	9.25	8.77	8.97
EnAd-CMV-ControlBiTE	8.23	8.45	8.87	8.51	8.7	9.5
EnAd-CMV-EpCAMBiTE	7.79	9.52	9.98	72.7	75.9	79
EnAd-SA-ControlBiTE	8.75	8.23	8.25	8.31	8.99	8.88
EnAd-SA-EpCAMBiTE	9.06	8.97	8.55	66.8	68.9	72.8
