# Supplementary material for: Oncolytic adenovirus expressing bispecific antibody targets T‐cell cytotoxicity in cancer biopsies
Source: EMBO Mol Med. 2017 Jun 20;9(8):1067–87. doi: 10.15252/emmm.201707567 (PMC5538299; doi:10.15252/emmm.201707567)
Supplement: Supplementary file 14 — Source Data for Figure 4 [file EMMM-9-1067-s012.zip › EMM_07567_Fig4_Source_data/Fig4F.pdf]

Treatment	Cytotoxicity (%)											
	CHO						CHO-EpCAM					
	no T-cells			with T-cells			no T-cells			with T-cells		
	1	2	3	1	2	3	1	2	3	1	2	3
Uninfected	-1.34	-1.94	-1.90	-3.28	-2.33	-3.44	4.22	7.25	5.54	2.02	6.33	7.86
EnAd	-1.46	-2.13	-2.13	-3.16	-2.09	-2.41	4.92	5.40	7.47	7.95	5.45	7.95
EnAd-CMV-ControlBiTE	-0.28	-0.16	0.16	-1.30	-1.74	-1.66	15.38	10.15	5.18	5.67	8.74	7.69
EnAd-CMV-EpCAMBiTE	-0.51	-0.99	-1.23	-2.65	-1.66	-0.43	4.79	2.46	5.54	38.01	39.32	39.06
EnAd-SA-ControlBiTE	-1.19	-0.83	-0.04	-2.61	-2.41	-1.62	5.14	3.73	1.63	7.21	4.22	5.58
EnAd-SA-EpCAMBiTE	1.50	-2.41	-1.94	-2.02	-2.69	-1.66	4.17	-0.26	-1.49	27.46	29.04	29.17
