# Supplementary material for: Oncolytic adenovirus expressing bispecific antibody targets T‐cell cytotoxicity in cancer biopsies
Source: EMBO Mol Med. 2017 Jun 20;9(8):1067–87. doi: 10.15252/emmm.201707567 (PMC5538299; doi:10.15252/emmm.201707567)
Supplement: Supplementary file 15 — Source Data for Figure 5 [file EMMM-9-1067-s013.zip › EMM_07567_Fig5_Source_data/Fig5C.pdf]

Time (h)										CD69-positive (%)
	Uninfected			EnAd			EnAd-CMV-ControlBiTE			EnAd-C
	1	2	3	1	2	3	1	2	3	1
0	14.7	15.3	15	14.7	15.3	15	14.7	15.3	15	14.7
24	8.77	9.42	12.2	11.6	10.3	11.3	11	9.52	8.41	59.8
48	10.7	14.3	7.88	7.63	7.55	5.83	9.12	6.4	6.94	72.2
96	12.3	11.6	13.4	13.5	16.2	13.1	15.2	14.7	13.8	64.7

CMV-EpCAMBiTE		EnAd-SA-ControlBiTE			EnAd-SA-EpCAMBiTE		
2	3	1	2	3	1	2	3
15.3	15	14.7	15.3	15	14.7	15.3	15
65.9	65.2	9.04	9.13	11.1	9.73	12.3	16.9
67.9	64.8	7.8	8.03	9.3	71.6	69.1	64.9
67.6	65.7	16	16.5	16.8	57.8	59.5	62.4
