# Supplementary material for: Oncolytic adenovirus expressing bispecific antibody targets T‐cell cytotoxicity in cancer biopsies
Source: EMBO Mol Med. 2017 Jun 20;9(8):1067–87. doi: 10.15252/emmm.201707567 (PMC5538299; doi:10.15252/emmm.201707567)
Supplement: Supplementary file 15 — Source Data for Figure 5 [file EMMM-9-1067-s013.zip › EMM_07567_Fig5_Source_data/Fig5D.pdf]

Time (h)										CD25-positive (%)
	Uninfected			EnAd			EnAd-CMV-ControlBiTE			EnAd-C
	1	2	3	1	2	3	1	2	3	1
0	0.7	0.68	0.66	0.7	0.68	0.66	0.7	0.68	0.66	0.7
24	1.07	1.27	1.53	1.19	1.36	1.09	0.93	0.89	0.97	1.17
48	2.3	2.14	1.68	2.22	1.97	1.97	3.52	2.03	1.78	30.2
96	3.25	3.84	3.34	2.71	3.8	3.37	2.41	3.21	3.08	42.2

CMV-EpCAMBiTE		EnAd-SA-ControlBiTE			EnAd-SA-EpCAMBiTE		
2	3	1	2	3	1	2	3
0.68	0.66	0.7	0.68	0.66	0.7	0.68	0.66
1.57	1.3	1.05	1.1	0.92	1.25	0.8	0.89
27.1	22.1	1.87	1.83	2.67	9.93	5.25	8.24
46.2	42.9	2.36	2.66	3.2	35.5	40.7	37.2
