# Supplementary material for: Oncolytic adenovirus expressing bispecific antibody targets T‐cell cytotoxicity in cancer biopsies
Source: EMBO Mol Med. 2017 Jun 20;9(8):1067–87. doi: 10.15252/emmm.201707567 (PMC5538299; doi:10.15252/emmm.201707567)
Supplement: Supplementary file 16 — Source Data for Figure 6 [file EMMM-9-1067-s014.zip › EMM_07567_Fig6_Source_data/Fig6A.pdf]

Treatment	EpCAM+ cells (%)								
	Patient 1			Patient 2			Patient 3		
	1	2	3	1	2	3	1	2	3
Untreated	43.40	148.38	108.23	73.90	140.33	85.76	59.13	132.73	108.16
Control BiTE	41.41	174.16	204.40	89.17	112.49	112.03	63.48	194.40	152.63
EpCAM BiTE	0.00	0.00	4.40	0.00	1.19	0.00	1.10	1.76	0.00
EnAd	91.27	134.12	82.66	98.24	108.72	110.87	66.10	153.68	137.99
EnAd-CMV-ControlBiTE	93.65	115.03	110.35	94.65	108.81	127.85	101.43	121.97	73.23
EnAd-CMV-EpCAMBiTE	0.00	0.00	0.00	0.87	0.69	1.92	0.00	0.00	0.94
EnAd-SA-ControlBiTE	63.07	160.02	100.05	90.27	103.68	113.78	97.99	108.63	68.73
EnAd-SA-EpCAMBiTE	0.00	2.83	3.88	1.34	0.00	1.62	0.00	0.00	0.00
