# Supplementary material for: Oncolytic adenovirus expressing bispecific antibody targets T‐cell cytotoxicity in cancer biopsies
Source: EMBO Mol Med. 2017 Jun 20;9(8):1067–87. doi: 10.15252/emmm.201707567 (PMC5538299; doi:10.15252/emmm.201707567)
Supplement: Supplementary file 16 — Source Data for Figure 6 [file EMMM-9-1067-s014.zip › EMM_07567_Fig6_Source_data/Fig6B.pdf]

Treatment	FAP+ cells (%)								
	Patient 1			Patient 2			Patient 3		
	1	2	3	1	2	3	1	2	3
Untreated	42.07	138.33	119.61	87.77	132.68	79.56	54.879	129.11	116.01
Control BiTE	51.52	136.16	139.44	82.76	88.723	90.84	63.932	153.16	128.5
EpCAM BiTE	34.23	103.09	106.41	117.2	136.55	119.08	81.17	210.39	133.15
EnAd	61.54	116.59	82.117	69.81	98.642	84.491	68.949	131.32	119.71
EnAd-CMV-ControlBiTE	68.09	86.724	92.477	72.75	93.798	103.96	90.198	59.123	50.909
EnAd-CMV-EpCAMBiTE	42.33	66.947	59.531	114.7	78.237	141.04	48.367	86.004	59.223
EnAd-SA-ControlBiTE	50.38	85.424	90.149	80.24	99.387	102.79	79.55	82.344	58.502
EnAd-SA-EpCAMBiTE	38.56	41.629	65.847	81.05	77.411	110.78	73.156	57.543	91.919
