# Supplementary material for: Oncolytic adenovirus expressing bispecific antibody targets T‐cell cytotoxicity in cancer biopsies
Source: EMBO Mol Med. 2017 Jun 20;9(8):1067–87. doi: 10.15252/emmm.201707567 (PMC5538299; doi:10.15252/emmm.201707567)
Supplement: Supplementary file 16 — Source Data for Figure 6 [file EMMM-9-1067-s014.zip › EMM_07567_Fig6_Source_data/Fig6C.pdf]

Treatment	CD25-positive (%)								
	Patient 1			Patient 2			Patient 3		
	1	2	3	1	2	3	1	2	3
Untreated	9.36	10.9	9.25	13.5	13.7	12.3	12.3	12	12.1
Control BiTE	8.6	10.2	11.6	13.2	12.9	12.7	13.8	13.3	14
EpCAM BiTE	72.8	76.7	80.6	87.8	87.8	86.7	87.2	88.5	87.1
EnAd	10	10.7	10.7	11.8	15.2	12.5	13.8	13.6	14.1
EnAd-CMV-ControlBiTE	8.57	8.54	8.71	10.6	10.3	9.95	10.3	9.76	11.7
EnAd-CMV-EpCAMBiTE	71.1	76.2	75.5	86.5	85.8	86.8	81.1	86.2	85.6
EnAd-SA-ControlBiTE	8.4	7.62	7.85	11.6	12.7	11.7	11.3	10.7	11
EnAd-SA-EpCAMBiTE	70.8	77.2	75	87.1	85.9	86.8	82.7	84.5	84.7
