# Supplementary material for: Oncolytic adenovirus expressing bispecific antibody targets T‐cell cytotoxicity in cancer biopsies
Source: EMBO Mol Med. 2017 Jun 20;9(8):1067–87. doi: 10.15252/emmm.201707567 (PMC5538299; doi:10.15252/emmm.201707567)
Supplement: Supplementary file 17 — Source Data for Figure 7 [file EMMM-9-1067-s015.zip › EMM_07567_Fig7_Source_data/Fig7D.pdf]

**Sample**	**CD69+CD25+ (%)**					
	Control BiTE			EpCAM BiTE		
	1	2	3	1	2	3
normal serum	1.5	1.27	1.33	68.4	67.6	67.5
A1	2.34	1.93	2.32	72	70	71.7
A2	1.44	1.1	1.15	71.5	70.9	71.3
A3	1.42	0.91	1.4	71.4	72	70.7
A6	1.58	1.82	1.61	72	71.7	72
A7	1.22	1.02	1.09	70.9	68.2	69.1
P4	0.78	0.89	0.75	66.1	66.7	69.5
P5	1.14	0.85	0.93	68.1	67.7	67.1
