# Supplementary material for: Oncolytic adenovirus expressing bispecific antibody targets T‐cell cytotoxicity in cancer biopsies
Source: EMBO Mol Med. 2017 Jun 20;9(8):1067–87. doi: 10.15252/emmm.201707567 (PMC5538299; doi:10.15252/emmm.201707567)
Supplement: Supplementary file 18 — Source Data for Figure 8 [file EMMM-9-1067-s016.zip › EMM_07567_Fig8_Source_data/Fig8B.pdf]

**Treatment**	**CD69+CD25+ (%)**					
	RPMI			Ascites fluid		
	1	2	3	1	2	3
Untreated	0.69	0.86	0.71	0.6	0.77	0.56
Control BiTE	0.5	0.53	0.71	0.52	0.82	0.69
EpCAM BiTE	24	28.3	33.2	40.4	36.2	33.3
