# Supplementary material for: Oncolytic adenovirus expressing bispecific antibody targets T‐cell cytotoxicity in cancer biopsies
Source: EMBO Mol Med. 2017 Jun 20;9(8):1067–87. doi: 10.15252/emmm.201707567 (PMC5538299; doi:10.15252/emmm.201707567)
Supplement: Supplementary file 18 — Source Data for Figure 8 [file EMMM-9-1067-s016.zip › EMM_07567_Fig8_Source_data/Fig8C.pdf]

Treatment	CD25+ (%)					
	RPMI			Ascites fluid		
	1	2	3	1	2	3
Untreated	9.4	6.41	7.14	16.6	11.9	15.2
control BiTE	9.96	7.29	7.33	16.6	14	12.5
EpCAM BiTE	85.9	85.7	85.5	94.4	94.9	95
EnAd	11.6	14.3	13.6	16.2	15.3	18
EnAd-CMV-controlBiTE	10.4	13.7	15.9	20.2	18.6	15.6
EnAd-CMV-EpCAMBiTE	88	88.7	90.8	95	94.2	94.3
EnAd-SA-controlBiTE	13.6	13.6	14	19.5	16.8	17.6
EnAd-SA-EpCAMBiTE	66.3	63.2	61.9	80	80.3	86.6
