# Supplementary material for: Oncolytic adenovirus expressing bispecific antibody targets T‐cell cytotoxicity in cancer biopsies
Source: EMBO Mol Med. 2017 Jun 20;9(8):1067–87. doi: 10.15252/emmm.201707567 (PMC5538299; doi:10.15252/emmm.201707567)
Supplement: Supplementary file 18 — Source Data for Figure 8 [file EMMM-9-1067-s016.zip › EMM_07567_Fig8_Source_data/Fig8D.pdf]

**Treatment**	**CD3+ cells**					
	RPMI			Ascites fluid		
	1	2	3	1	2	3
Untreated	3816.74	3671.84	4510.53	4082.79	4209.09	4198.61
control BiTE	4396.04	4900.18	4395.58	4281.58	4080.59	4253.06
EpCAM BiTE	4596.22	4354.52	5214.65	9877.55	13230.74	13140.81
EnAd	5809.33	6442.77	5448.05	5442.96	4738.57	6034.09
EnAd-CMV-controlBiTE	6203.43	4782.84	5885.32	5028.19	6319.53	4806.10
EnAd-CMV-EpCAMBiTE	3830.24	2981.79	3191.48	11756.15	10228.90	11423.92
EnAd-SA-controlBiTE	5244.18	5406.77	5839.33	4584.25	4890.33	5786.44
EnAd-SA-EpCAMBiTE	1280.03	1167.78	1769.01	2247.09	4663.40	4903.38
