# Supplementary material for: Oncolytic adenovirus expressing bispecific antibody targets T‐cell cytotoxicity in cancer biopsies
Source: EMBO Mol Med. 2017 Jun 20;9(8):1067–87. doi: 10.15252/emmm.201707567 (PMC5538299; doi:10.15252/emmm.201707567)
Supplement: Supplementary file 18 — Source Data for Figure 8 [file EMMM-9-1067-s016.zip › EMM_07567_Fig8_Source_data/Fig8E.pdf]

Treatment	EpCAM+ cells					
	RPMI			Ascites fluid		
	1	2	3	1	2	3
Untreated	31625.8	27978.0	35027.4	36475.4	39029.8	35452.2
control BiTE	31556.2	36794.6	30702.3	34483.8	34679.3	35292.6
EpCAM BiTE	92.3	71.9	78.4	383.7	512.2	873.6
EnAd	44649.6	31940.9	29056.3	32366.6	39466.8	40704.6
EnAd-CMV-controlBiTE	36369.1	29462.3	33424.2	36613.7	43474.3	42086.9
EnAd-CMV-EpCAMBiTE	1653.9	1717.6	1274.3	1097.5	808.2	1021.6
EnAd-SA-controlBiTE	37441.0	35316.5	37371.7	32785.7	29949.4	47237.8
EnAd-SA-EpCAMBiTE	686.2	769.7	1059.3	7874.2	8577.0	4265.5
