# Supplementary material for: Oncolytic adenovirus expressing bispecific antibody targets T‐cell cytotoxicity in cancer biopsies
Source: EMBO Mol Med. 2017 Jun 20;9(8):1067–87. doi: 10.15252/emmm.201707567 (PMC5538299; doi:10.15252/emmm.201707567)
Supplement: Supplementary file 19 — Source Data for Figure 9 [file EMMM-9-1067-s017.zip › EMM_07567_Fig9_Source_data/Fig9A.pdf]

Treatment	CD3+ cells (fold)												
	Patient 1			Patient 2			Patient 3			Patient 4			P
	1	2	3	1	2	3	1	2	3	1	2	3	1
Untreated	0.98	1.01	1.01	0.90	1.11	0.99	0.84	0.95	1.21	1.03	1.03	0.94	0.54
Control BiTE	1.03	0.98	1.02	0.81	0.80	1.18	0.75	1.02	0.95	1.01	1.11	1.02	0.48
EpCAM BiTE	2.37	3.18	3.16	1.63	1.53	1.80	26.16	42.85	30.22	6.55	6.35	7.16	1.43
EnAd	1.31	1.14	1.45	1.01	1.06	1.12	1.25	1.28	1.35	1.06	1.07	0.81	0.95
EnAd-CMV-controlBiTE	1.21	1.52	1.15	0.94	0.96	1.07	1.10	1.76	1.31	0.93	1.16	1.05	1.04
EnAd-CMV-EpCAMBiTE	2.82	2.46	2.74	1.41	1.80	1.47	17.58	17.82	46.04	6.80	6.73	6.06	1.99
EnAd-SA-controlBiTE	1.10	1.17	1.39	0.90	1.09	1.10	1.01	1.14	1.23	0.89	1.10	1.15	1.38
EnAd-SA-EpCAMBiTE	0.54	1.12	1.18	0.94	1.45	1.23	10.22	6.60	18.60	3.14	3.64	2.66	1.81

atient 5		Patient 6			Patient 7		
2	3	1	2	3	1	2	3
1.04	1.42	1.04	1.05	0.90	1.08	0.97	0.94
0.94	1.33	1.34	1.51	1.03	1.07	1.05	1.01
2.31	1.97	3.66	4.18	4.30	3.06	2.25	2.39
1.53	1.38	1.14	1.01	0.99	0.81	0.84	0.93
1.56	1.45	1.27	1.17	1.13	1.29	1.35	1.68
3.01	3.20	2.76	3.39	2.78	2.15	2.20	2.41
1.46	1.28	1.34	1.16	1.37	1.42	1.50	1.65
2.62	2.64	4.05	3.62	4.78	2.09	2.75	1.86
