# Supplementary material for: Oncolytic adenovirus expressing bispecific antibody targets T‐cell cytotoxicity in cancer biopsies
Source: EMBO Mol Med. 2017 Jun 20;9(8):1067–87. doi: 10.15252/emmm.201707567 (PMC5538299; doi:10.15252/emmm.201707567)
Supplement: Supplementary file 19 — Source Data for Figure 9 [file EMMM-9-1067-s017.zip › EMM_07567_Fig9_Source_data/Fig9B.pdf]

| **Treatment** | **CD25-positive (%)** | | | | | | | | | | | | |
|---|---|---|---|---|---|---|---|---|---|---|---|---|---|
| | Patient 1 | | | Patient 2 | | | Patient 3 | | | Patient 4 | | | P |
| | 1 | 2 | 3 | 1 | 2 | 3 | 1 | 2 | 3 | 1 | 2 | 3 | 1 |
| Untreated | 16.6 | 11.9 | 15.2 | 20.0 | 19.3 | 19.9 | 15.5 | 11.2 | 10.5 | 26.0 | 25.7 | 26.2 | 25.6 |
| Control BiTE | 16.6 | 14.0 | 12.5 | 17.6 | 20.4 | 19.4 | 10.0 | 11.2 | 11.9 | 29.7 | 27.0 | 27.1 | 27.1 |
| EpCAM BiTE | 94.4 | 94.9 | 95.0 | 74.0 | 77.3 | 77.5 | 94.5 | 96.2 | 96.4 | 92.1 | 93.0 | 92.9 | 94.5 |
| EnAd | 16.2 | 15.3 | 18.0 | 24.9 | 19.4 | 18.2 | 10.6 | 20.0 | 13.5 | 23.1 | 24.4 | 24.5 | 38.2 |
| EnAd-CMV-controlBiTE | 20.2 | 18.6 | 15.6 | 21.5 | 20.2 | 19.9 | 9.2 | 14.5 | 21.0 | 27.0 | 25.9 | 25.6 | 40.0 |
| EnAd-CMV-EpCAMBiTE | 95.0 | 94.2 | 94.3 | 53.9 | 51.0 | 51.9 | 34.6 | 22.7 | 21.5 | 81.3 | 77.9 | 80.3 | 95.0 |
| EnAd-SA-controlBiTE | 19.5 | 16.8 | 17.6 | 18.7 | 20.2 | 18.5 | 12.4 | 11.2 | 10.6 | 23.1 | 24.6 | 28.4 | 45.2 |
| EnAd-SA-EpCAMBiTE | 80.0 | 80.3 | 86.6 | 52.1 | 55.9 | 51.1 | 89.5 | 60.7 | 84.1 | 65.8 | 68.8 | 67.0 | 91.9 |

| atient 5 | | Patient 6 | | | Patient 7 | | |
|---|---|---|---|---|---|---|---|
| 2 | 3 | 1 | 2 | 3 | 1 | 2 | 3 |
| 27.8 | 37.6 | 19.6 | 20.9 | 18.0 | 5.1 | 5.3 | 5.3 |
| 32.1 | 32.7 | 25.4 | 27.9 | 20.2 | 5.1 | 5.5 | 6.2 |
| 94.5 | 94.9 | 77.2 | 81.8 | 78.8 | 49.8 | 55.1 | 55.0 |
| 41.1 | 29.5 | 23.9 | 17.3 | 20.2 | 5.2 | 4.4 | 4.4 |
| 36.1 | 38.7 | 26.0 | 19.7 | 17.3 | 5.1 | 5.1 | 5.3 |
| 92.6 | 94.2 | 66.8 | 60.2 | 64.1 | 31.6 | 27.8 | 31.2 |
| 41.0 | 41.9 | 18.6 | 19.1 | 23.9 | 5.4 | 5.1 | 5.2 |
| 92.9 | 93.0 | 67.8 | 64.9 | 63.0 | 11.7 | 17.4 | 19.1 |
