# Supplementary material for: Oncolytic adenovirus expressing bispecific antibody targets T‐cell cytotoxicity in cancer biopsies
Source: EMBO Mol Med. 2017 Jun 20;9(8):1067–87. doi: 10.15252/emmm.201707567 (PMC5538299; doi:10.15252/emmm.201707567)
Supplement: Supplementary file 19 — Source Data for Figure 9 [file EMMM-9-1067-s017.zip › EMM_07567_Fig9_Source_data/Fig9C.pdf]

Treatment												EpCAM+ cells (%)
	Patient 1			Patient 2			Patient 3			Patient 4		
	1	2	3	1	2	3	1	2	3	1	2	3
Untreated	98.6	105.5	95.9	88.8	106.4	104.8	87.1	106.4	106.5	76.6	113.7	109.7
Control BiTE	93.2	93.8	95.4	122.7	89.1	169.1	90.9	95.6	107.6	123.5	103.8	97.3
EpCAM BiTE	1.0	1.4	2.4	21.5	22.2	19.3	2.9	2.1	4.9	5.8	7.2	4.7
EnAd	87.5	106.7	110.1	72.9	109.7	109.6	70.6	118.7	87.2	88.9	112.7	76.8
EnAd-CMV-controlBiTE	99.0	117.5	113.8	86.5	131.0	122.7	98.6	103.7	95.7	84.3	105.1	114.3
EnAd-CMV-EpCAMBiTE	3.0	2.2	2.8	28.1	26.4	23.7	35.3	42.7	17.1	4.3	7.4	7.1
EnAd-SA-controlBiTE	88.6	81.0	127.7	92.4	116.4	97.7	87.0	99.8	101.9	66.7	127.6	118.9
EnAd-SA-EpCAMBiTE	21.3	23.2	11.5	11.0	19.0	12.1	29.4	46.6	23.7	3.3	10.2	3.9

Patient 5			Patient 6			Patient 7		
1	2	3	1	2	3	1	2	3
65.9	93.0	151.1	88.1	84.6	127.3	109.0	89.6	101.5
74.5	111.3	171.6	106.1	99.5	105.6	68.2	84.0	104.1
1.0	2.8	1.5	5.6	21.3	9.5	6.8	4.0	2.0
76.4	149.0	131.8	71.6	97.5	127.6	63.7	57.3	64.1
106.2	159.7	142.2	84.7	113.6	104.4	58.1	117.3	64.6
1.9	6.4	4.1	15.2	16.4	8.0	5.1	3.1	2.6
133.8	175.3	132.9	119.3	95.0	112.6	60.3	56.8	61.1
15.9	13.5	12.8	26.4	15.2	31.3	24.2	11.8	4.1
