# Supplementary material for: Oncolytic adenovirus expressing bispecific antibody targets T‐cell cytotoxicity in cancer biopsies
Source: EMBO Mol Med. 2017 Jun 20;9(8):1067–87. doi: 10.15252/emmm.201707567 (PMC5538299; doi:10.15252/emmm.201707567)
Supplement: Supplementary file 19 — Source Data for Figure 9 [file EMMM-9-1067-s017.zip › EMM_07567_Fig9_Source_data/Fig9E.pdf]

Cytokine									Quantity (pg
		Untreated			Control BiTE			EpCAM BiTE	
	1	2	3	1	2	3	1	2	3
IL-5	3.36	3.43	3.82	3.75	3.9	3.33	66.36	19.95	62.96
IL-13	<3.41	<3.41	3.73	3.67	3.99	<3.41	75.49	36.5	61.64
IL-2	5.03	5.15	8.11	6.8	7.51	6.95	5.03	3.84	4.81
IL-6	9396.85	11107.29	17313.22	17473.68	14130.39	10702.58	34165.81	26948.44	33624.63
IL-9	3.99	3.95	4.29	3.7	4.26	3.77	25.92	12.76	28.68
IL-10	9.85	9.94	18.53	18.7	15.74	12.27	33.8	13.89	34.8
IFNg	4.74	5.26	4.74	6.33	7.14	7.29	6040.81	2059.77	2541.78
TNFa	4.13	4.43	5.07	6.12	5	4.8	91.12	37.81	80.72
IL-17A	4.92	4.92	5.84	7.17	6	5.29	508.42	169.88	211.31
IL-17F	<3.49	<3.49	3.5	3.89	3.5	<3.49	161.65	52.02	93.99
IL-4	5.25	5.07	5.64	6.05	5.95	6.05	8.1	4.76	7.44
IL-21	42.92	52.03	90.14	96.64	82.99	60.13	228.91	97.76	184.1
IL-22	10.02	10.02	10.91	14.49	13.8	13.13	93.46	27.57	61.49

Cytokine									Quantity (fold-
		Untreated			Control BiTE			EpCAM BiTE	
	1	2	3	1	2	3	1	2	3
IL-5	0.95	0.97	1.08	1.06	1.10	0.94	18.80	5.65	17.84
IL-13	0.97	0.97	1.06	1.04	1.13	0.97	21.45	10.37	17.51
IL-2	0.82	0.84	1.33	1.11	1.23	1.14	0.82	0.63	0.79
IL-6	0.75	0.88	1.37	1.39	1.12	0.85	2.71	2.14	2.67
IL-9	0.98	0.97	1.05	0.91	1.04	0.92	6.35	3.13	7.03
IL-10	0.77	0.78	1.45	1.46	1.23	0.96	2.65	1.09	2.73
IFNg	1.16	1.28	1.16	1.54	1.74	1.78	1473.37	502.38	619.95
TNFa	0.91	0.98	1.12	1.35	1.10	1.06	20.07	8.33	17.78
IL-17A	0.94	0.94	1.12	1.37	1.15	1.01	97.21	32.48	40.40
IL-17F	1.00	1.00	1.00	1.11	1.00	1.00	46.32	14.91	26.93
IL-4	0.99	0.95	1.06	1.14	1.12	1.14	1.52	0.89	1.40
IL-21	0.70	0.84	1.46	1.57	1.35	0.97	3.71	1.58	2.98
IL-22	1.00	1.00	1.09	1.45	1.38	1.31	9.33	2.75	6.14

/mL)

EnAd			EnAd-CMV-ControlBiTE			EnAd-SA-ControlBiTE		
1	2	3	1	2	3	1	2	3
4.39	3.29	3.2	3.04	3.13	3.6	26.21	29.61	31.48
4.96	3.42	<3.41	<3.41	<3.41	3.86	48.7	42.37	57.77
13.7	25.63	22.28	7.76	9.56	31.78	6.09	5.57	5.32
7441.83	8094.94	6345.25	3422.84	5457.5	11210.79	34165.81	34165.81	34165.81
<3.70	4.4	3.77	<3.70	3.99	4.7	34.48	28.39	26.18
10.85	8.44	8.73	5.66	6.21	13.64	94.26	100.66	58.51
26.58	53.62	62.34	52.01	32.4	150.99	2779.94	2658.56	5710.51
4.31	4.37	3.86	3.55	4.8	5.72	24.75	24.16	37.38
5.29	5.21	5.06	<4.19	5.52	15.82	75.52	102.24	147.55
<3.49	<3.49	<3.49	<3.49	<3.49	3.69	20.89	14.22	50.25
5.54	5.54	5.35		5.07	5.95	7.96	7.44	7.19
37.71	47.32	33.47	18.45	30.52	68.27	201.46	215.88	191.66
10.31	8.63	9.17	9.17	12.47	12.15	59.13	43.51	76.86

change)

EnAd			EnAd-CMV-ControlBiTE			EnAd-SA-ControlBiTE		
1	2	3	1	2	3	1	2	3
1.24	0.93	0.91	0.86	0.89	1.02	7.42	8.39	8.92
1.41	0.97	0.97	0.97	0.97	1.10	13.84	12.04	16.41
2.25	4.20	3.65	1.27	1.57	5.21	1.00	0.91	0.87
0.59	0.64	0.50	0.27	0.43	0.89	2.71	2.71	2.71
0.91	1.08	0.92	0.91	0.98	1.15	8.45	6.96	6.42
0.85	0.66	0.68	0.44	0.49	1.07	7.38	7.88	4.58
6.48	13.08	15.20	12.69	7.90	36.83	678.03	648.43	1392.81
0.95	0.96	0.85	0.78	1.06	1.26	5.45	5.32	8.23
1.01	1.00	0.97	0.80	1.06	3.02	14.44	19.55	28.21
1.00	1.00	1.00	1.00	1.00	1.06	5.99	4.07	14.40
1.04	1.04	1.01	0.00	0.95	1.12	1.50	1.40	1.35
0.61	0.77	0.54	0.30	0.49	1.11	3.27	3.50	3.11
1.03	0.86	0.92	0.92	1.24	1.21	5.90	4.34	7.67
